# Heating-Induced Switching to Hierarchical Liquid Crystallinity Combining Colloidal and Molecular Order in Zwitterionic Molecules

**DOI:** 10.1021/acsomega.3c04914

**Published:** 2023-10-12

**Authors:** Lotta Gustavsson, Zhong-Peng Lv, Tomy Cherian, Wille Seppälä, Ville Liljeström, Bo Peng, Simo Huotari, Patrice Rannou, Olli Ikkala

**Affiliations:** † Department of Applied Physics, Aalto University, Puumiehenkuja 2, FI-00076 Espoo, Finland; ‡ Nanomicroscopy Center, Aalto University, Puumiehenkuja 2, FI-00076 Espoo, Finland; § Department of Physics, University of Helsinki, P.O. Box 64, FI-00014 Helsinki, Finland; ∥ Université Grenoble Alpes, Université Savoie Mont-Blanc, CNRS, Grenoble INP, LEPMI, 38000 Grenoble, France

## Abstract

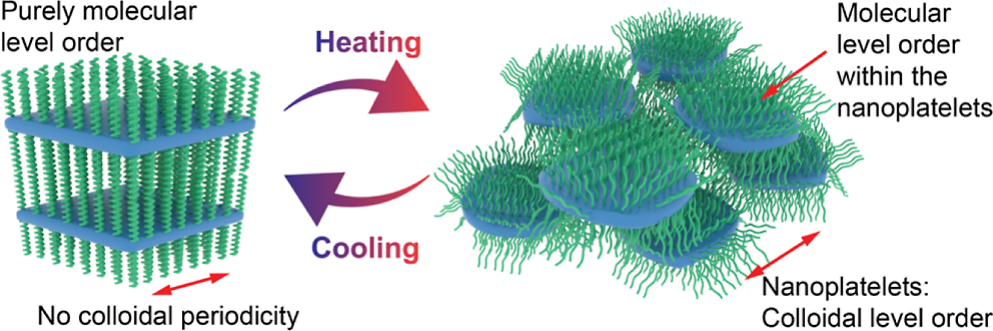

Hierarchical self-assemblies of soft matter involving triggerable or switchable structures at different length scales have been pursued toward multifunctional behaviors and complexity inspired by biological matter. They require several and balanced competing attractive and repulsive interactions, which provide a grand challenge in particular in the “bulk” state, i.e., in the absence of plasticizing solvents. Here, we disclose that zwitterionic bis-*n*-tetradecylphosphobetaine, as a model compound, shows a complex thermally switchable hierarchical self-assembly in the solvent-free state. It shows polymorphism and heating-induced reversible switching from low-temperature molecular-level assemblies to high-temperature hierarchical self-assemblies, unexpectedly combining colloidal and molecular self-assemblies, as inferred by synchrotron small-angle X-ray scattering (SAXS). The high-temperature phase sustains birefringent flow, indicating a new type of hierarchical thermotropic liquid crystallinity. The high-temperature colloidal-level SAXS reflections suggest indexation as a 2D oblique pattern and their well-defined layer separation in the perpendicular direction. We suggest that the colloidal self-assembled motifs are 2D nanoplatelets formed by the lateral packing of the molecules, where the molecular packing frustration between the tightly packed zwitterionic moieties and the coiled alkyl chains demanding more space limits the lateral platelet growth controlled by the alkyl stretching entropy. An indirect proof is provided by the addition of plasticizing ionic liquids, which relieve the ionic dense packings of zwitterions, thus allowing purely smectic liquid crystallinity without the colloidal level order. Thus, molecules with a simple chemical structure can lead to structural hierarchy and tunable complexity in the solvent-free state by balancing the competing long-range electrostatics and short-range nanosegregations.

## 1 Introduction

A plethora of classic soft matter self-assemblies involving single-length scale order have already been explored based on block copolymers, block oligomers, liquid crystals, and surfactants.^1−8^ Beyond them, hierarchical self-assemblies and superlattices of soft matter involving order at several length scales attract growing attention.^9−18^ An obvious motivation is that hierarchies can facilitate multifunctional materials, as different functions can require separate length scales. More conceptually, progressive levels of hierarchies can promote understanding of biomolecular complexities.^19^ In artificial soft matter, hierarchical self-assemblies have been achieved by engineering different competing structural packing motifs. Selected examples are given by various architectures in multiblock copolymers, combinations of mesogenic and flexible block packings in liquid crystalline polymers, or combinations of different length-scale structural motifs such as supramolecular complexation of block copolymers with surfactants or liquid crystals.^9−18^ Toward solvent-free low-molecular-weight soft matter, achieving hierarchies becomes especially challenging, requiring higher, still competitive repulsions (χ) between the blocks. Architected liquid crystals with shape-persistent mesogens in combination with tailored molecular topologies and supramolecular interactions have allowed complex self-assemblies.^20,21^ On the other hand, ionic interactions within low-molecular-weight molecular self-assemblies have recently become appreciated.^11,22−31^ Therein, a particular case deals with zwitterions with bound positive and negative charges in the compounds, i.e., involving no counterions.^32−38^ In such approaches, the long-range electrostatic interactions of ionic groups facilitate liquid crystallinity instead of the shape-persistent mesogens.

**Figure 1:**
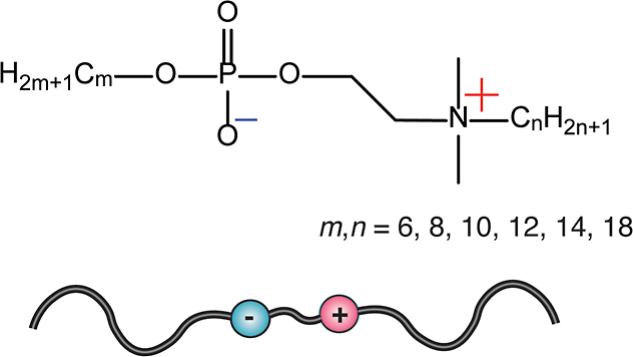
Chemical structures of the bis-*n*-alkylphosphobetaine zwitterionic molecules denoted as C_*m*_–C_*n*_ using different alkyl tail lengths studied in the solvent-free case. Herein, the focus is at C_14_–C_14_.

Here, we explore whether combining competing long-range electrostatic and short-range alkyl packing interactions in the solvent-free “bulk” state can provide routes for hierarchical self-assemblies. Alternatively, can charged small-molecular-weight molecules with seemingly simple structures lead to self-assembled complexity? i.e., how can molecular simplicity turn into structural complexity? We explore alkylated zwitterionic molecules in the solvent-free state (Figure 1), where the highly polar zwitterionic group mediates long-range electrostatic interactions competing with repulsive nonpolar alkyl tails, facilitating local steric packing and high-χ nanosegregations. Their aqueous behavior has been reported,^39,40^ but the solvent-free behavior remains unreported and turns out to be interesting and complex. We first screened temperature-dependent self-assemblies of zwitterionic bis-*n*-alkylphosphobetaines with different *n*-alkyl tail lengths from *n*-hexyl to *n*-octadecyl denoted as C_*m*_–C_*n*_ in the solvent-free state (Figure 1 and Supporting Information). Based on this compositional screening, we set here the focus for tetradecyl tails (C_14_–C_14_). The properties are studied using synchrotron X-ray techniques (SAXS/WAXS), polarized optical microscopy (POM), differential scanning calorimetry (DSC), temperature-dependent Fourier-transform infrared spectroscopy (FTIR) and electrochemical impedance spectroscopy (EIS).

## 2 Results and Discussion

### 2.1 Materials Preparation and Basic Characterization

The syntheses and characterizations of alkylated zwitterionic bis-*n*-tetradecylphosphobetaines C_14_–C_14_ are described in Methods and in Supporting Information Section S1, according to refs (39) and (40), where their aqueous behavior was explored, in contrast to the solvent-free assemblies herein. Thermogravimetric analysis (TGA) suggests the onset of the degradation close to the clearing points at ca. 200 °C (see Supporting Information Section S2). POM manifests birefringence with increasing heating-induced fluidity (Supporting Information Section S3). The upper limit for detailed thermal scans was thus set at 160 °C to limit degradation. The molecules are hygroscopic, and despite the drying efforts, it is likely that residual water exists in the molecular structure, both absorbed and as water of crystallization, as the measurements are mainly performed in ambient conditions.

### 2.2 Self-Assembly

**Figure 2:**
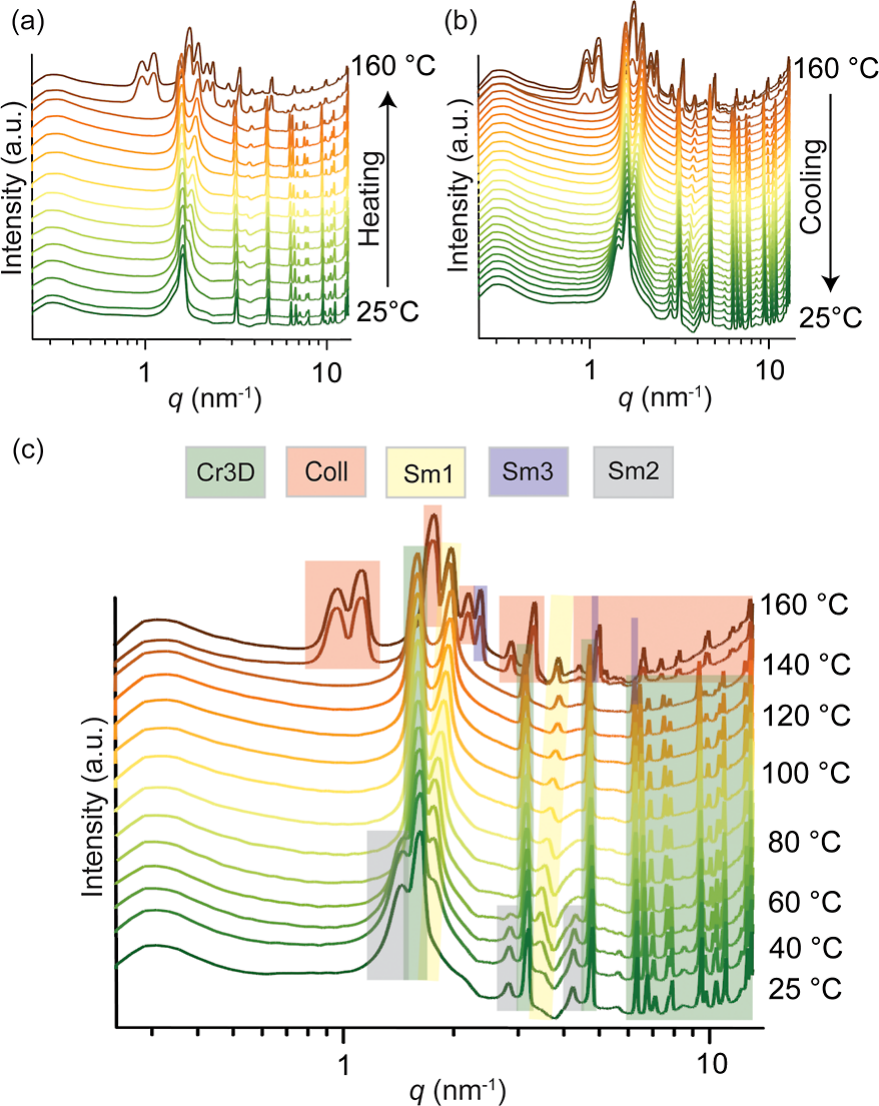
Temperature-dependent small-angle X-ray scattering (SAXS) 1D profiles of C_14_–C_14_ recorded during heating (a) and cooling (b). (c) For the sake of clarity in the subsequent discussion, the reflections allowing common indexations are denoted using different colors. The patterns are illustrated herein during cooling.

X-ray scattering patterns of C_14_–C_14_ show a particularly complex set of reflections and phase coexistences as a function of temperature (Figure 2a,b). The most striking observation is that while at low temperatures all reflections are observed at high scattering vector (*q*) magnitudes, signaling purely molecular level order, new additional reflections at much lower *q*-values are observed at high temperatures (Figure 2). This is observed reversibly upon heating and cooling (Figure 2a,b). Such low *q*-value reflections indicate the emergence of an additional order at several nanometer-length scales, i.e., at the colloidal-level periodicity. Therefore, a first qualitative conclusion can be drawn that, upon heating, a hierarchical self-assembly combining both colloidal and molecular length scales takes place, whereas at lower temperatures there is only molecular scale order. At this point, such a finding is surprising, as the colloidal length scale assembly cannot be directly predicted from the molecular structure and the molecular dimensions, indicating complexity. Also, it is, at first sight, somewhat counterintuitive that, upon heating, a new order is created.

**Figure 3:**
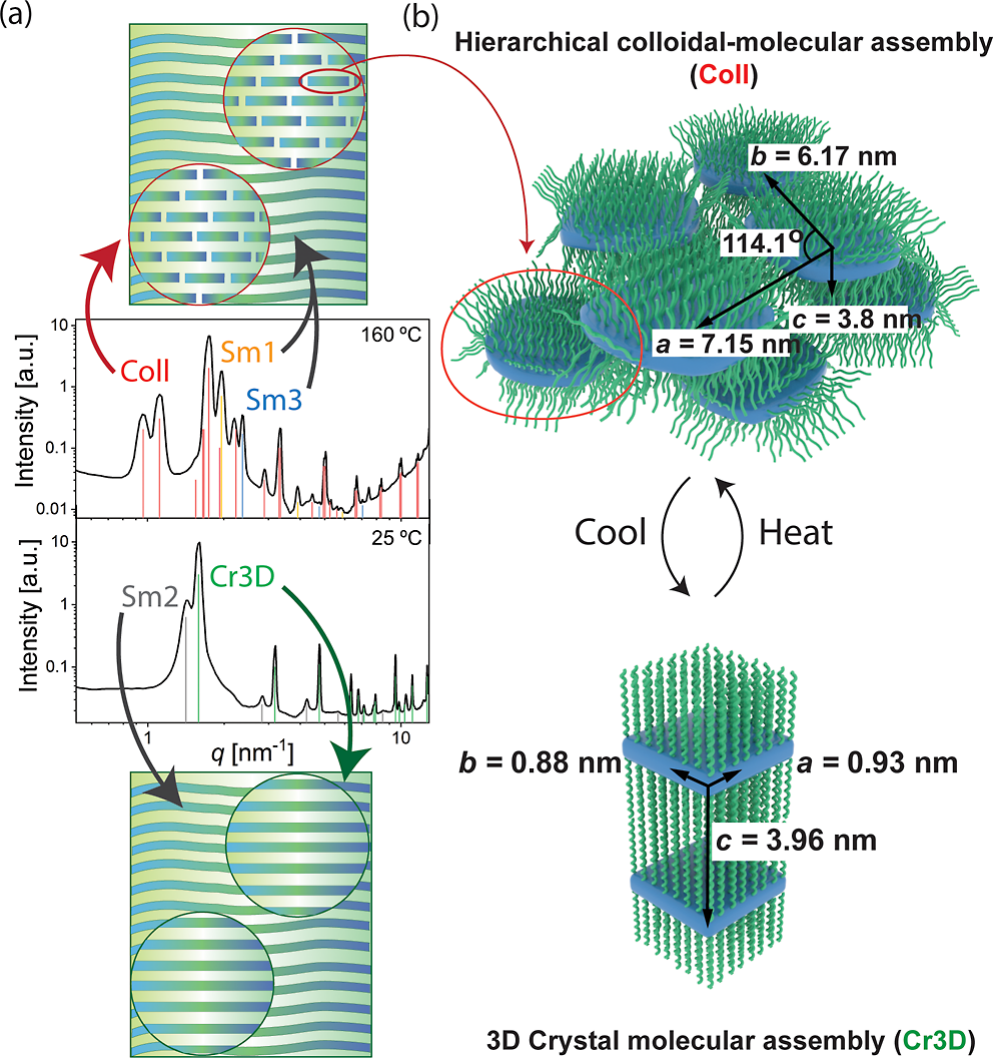
Phase behavior of C_14_–C_14_. (a) 1D SAXS profiles at 160 and 25 °C show the observed reflections indexed for the observed structures: 3D crystalline structure (Cr3D, green), hierarchical colloidal-molecular assembly (Coll, red), and the coexistent lamellar structures (Sm1, Sm2, and Sm3). The overviews of their coassembly are presented, corresponding to the SAXS graphs at 25 and 160 °C. (b) Reversible thermal transition involving the 3D crystal molecular assembly (Cr3D) and hierarchical colloidal-molecular assembly (Coll).

The reflections are next grouped to allow indexation; see Figure 2c (see Supporting Information Section S4 for details). At all temperatures, the reflections can be indexed based on several coexisting layered phases. Below ca. 145 °C, the overruling phase is a molecular self-assembly involving a 3D-crystalline layered phase denoted as Cr3D, whose reflections are illustrated in green in Figure 2c and Figure 3a. At room temperature, it can be indexed as an orthorhombic lattice with unit cell parameters of *a* = 0.93 nm, *b* = 0.88 nm, *c* = 3.96 nm, and *a* = β = γ = 90°, where *c* denotes the lamellar periodicity between the zwitterionic layers (see Figure 3b). A slight tilting upon increasing temperature is observed, as seen by gradual shifts in the crystallographic parameters (Supporting Information, Section S4).

There exists also another lamellar structure observed only upon cooling from high temperatures below ca. 60 °C down to room temperature (indicated as gray color in Figures 2b and 3a, denoted as Sm_2_). Its equidistant reflections at *q* = 1.44 (001), 2.80 (002), and 4.30 (003) nm^–1^ indicate a periodicity of 4.45 nm. This is larger than the length of the C_14_–C_14_ molecule (4.09 nm), therefore suggesting a nonoptimal packing of the molecules. Observed only upon cooling scan, this structure is monotropic and sacrificial, eventually leading to the growth of the Cr3D phase.

Still another coexisting structure can be identified at all temperatures, as indicated in yellow in the SAXS curves in Figure 2c. At 120 °C, such reflections are observed at *q* = 1.91, 3.82, and 5.65 (weak) nm^–1^, corresponding to 001, 002, and 003 reflections. Their equidistancy indicates a lamellar structure, denoted henceforth as Sm_1_ (yellow). The periodicity shifts from 3.6 to 3.2 nm upon heating from 55 to 145 °C, suggesting a gradually increasing degree of interdigitation of *n*-tetradecyl chains upon heating as the tails become gradually more molten.

**Figure 4:**
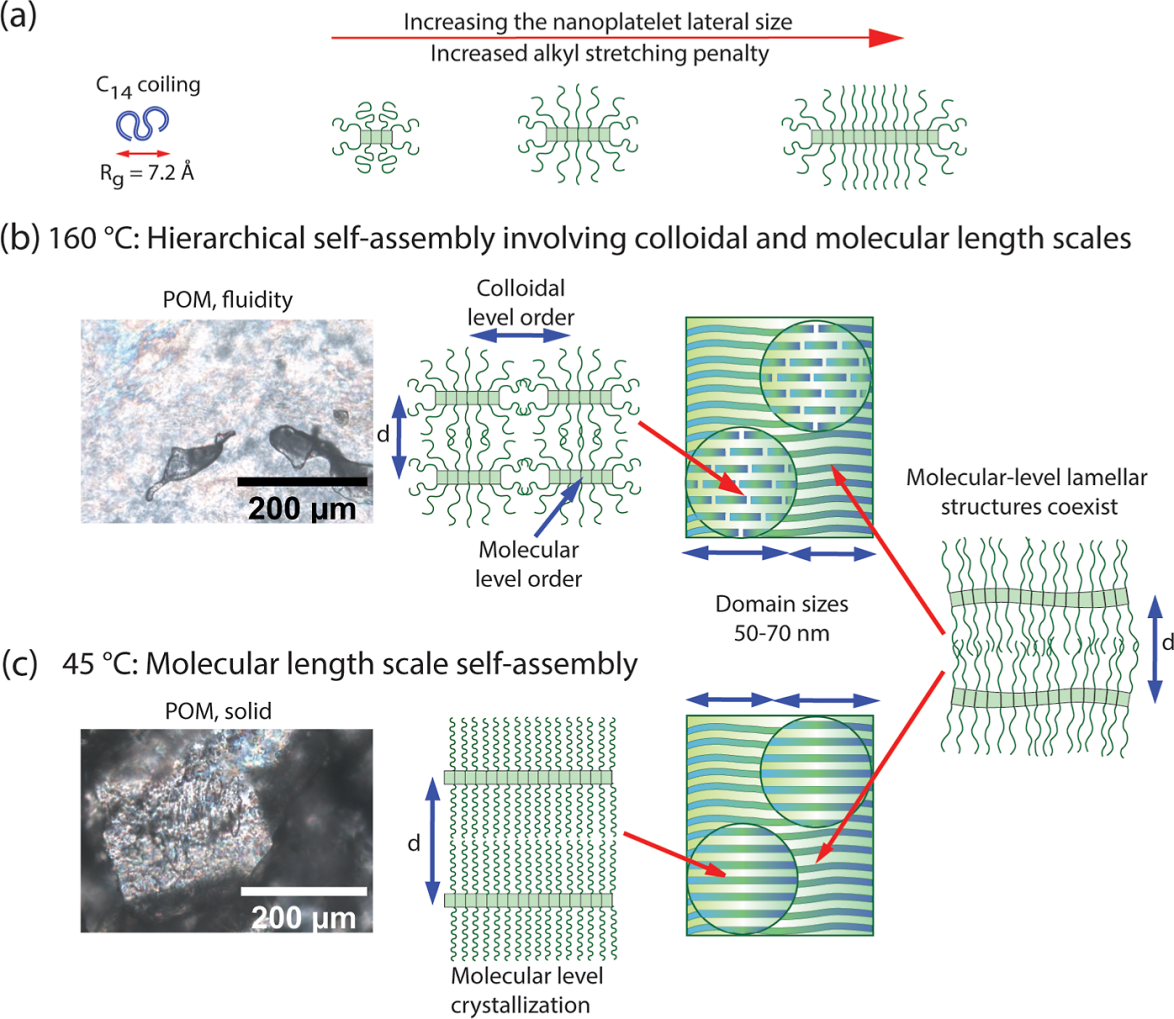
Suggested schemes for the complex coexistent self-assemblies based on X-ray patterns and POM microphotographs. (a) Suggestion how the alkyl chain stretching penalty in combination with the zwitterionic tight packing can limit the lateral growth of the 2D nanoplatelets. (b) At 160 °C, a hierarchical self-assembly is observed, consisting of both molecular and colloidal-level length scales. The colloidal-level 2D nanoplatelets are well-defined in the lateral dimension, leading to their oblique assembly. The nanoplatelets have also an internal molecular-level order, thus showing hierarchical order. Molecular smectic self-assembly coexists with the hierarchical superstructure. The material shows shear flow at 160 °C. (c) At 45 °C, the birefringent solid material corresponds to a molecular-level self-assembly, mainly consisting of Cr3D but coexisting with other lamellar structures.

Passing above 145 °C marks a fundamental change in the SAXS reflections, as new reflections at distinctly lower *q*-values are observable (marked as red in Figure 2c). Indexation is allowed based on two groups, i.e., 100, 010, 110, 020, 300, 040, 330, 050, and 060 according to 2D planar order, as well as in the perpendicular directions as 001, 002, 003, 004, 005, 006, and 007. The large number of such reflections indicates particularly well-defined structures (even if allowing birefringent fluidity, see later in connection to Figure 4). The first set of reflections suggests a well-developed oblique 2D colloidal order with nanometric lattice parameters *a* = 7.15 nm, *b* = 6.17 nm, and γ = 114.1°. This finding is subtle: it means that at high temperatures, new self-assembled colloidal-level structural units are formed in 2D beyond the molecular-level units to allow colloidal-level assemblies. It poses the question: what are these 2D colloidal units that allow such well-defined periodicity, as they cannot be directly inferred from the molecular structures? This question will be considered later. In the perpendicular direction, the periodicity is well-defined at *c* = 3.8 nm between the colloidal platelets. Additionally, at higher *q*-values, a wealth of other reflections are observed, thus indicating molecular order within the 2D nanoplatelets. Thus, the combination of colloidal and molecular scales suggests that the order is hierarchical, involving colloidal and molecular length scales.

Above 145 °C, yet another lamellar structure is found, i.e., Sm_3_. It has a short *d*-spacing of 2.67 nm, therefore suggesting a strongly interdigitated structure. At elevated temperatures, this may be expected as the thermal effects weaken the electrostatic interactions, leading to expansion in the lateral dimension.

POM shows that below 145 °C the materials are solid birefringent, agreeing with the crystallinity, whereas above 145 °C, birefringent fluidity is observed upon shearing (Figure 4, see also Figures S5 and S6). This suggests that the hierarchical assembly combining colloidal and molecular assemblies at high temperatures shows a new type of hierarchical colloidal/molecular-level liquid crystallinity. It suggests that the colloidal platelet layers are only weakly correlated with each other, and in spite of the high complexity, the high temperature phase can be denoted as a liquid crystal with a hierarchical structure. Previously, weakly correlated fluid layers have been discussed in the context of hexatic-B liquid crystals.^41^ On the other hand, studies involving ionic amphiphiles often involve soft crystalline phases, but their structures have only rarely been reported.^24,28,42,43^

**Figure 5:**
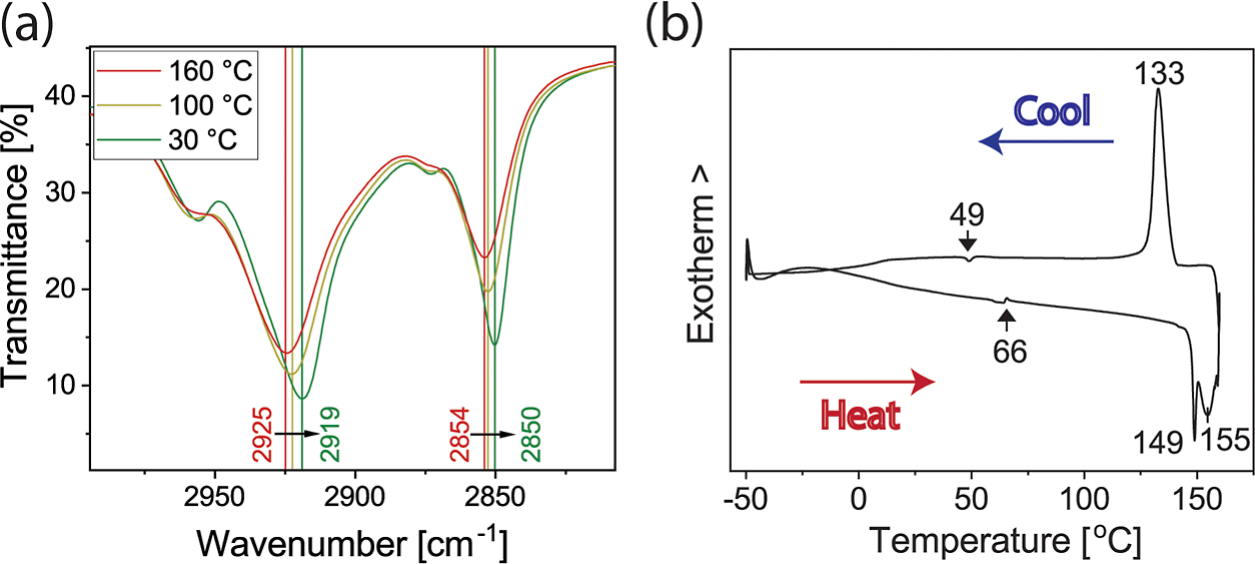
(a) Temperature-dependent FTIR spectra upon cooling of C_14_–C_14_. The shifts in the C–H stretching vibrations correspond to the transition of the alkyl tails from the molten to the crystalline state. (b) DSC thermograms of C_14_–C_14_. The main features deal with the transition from low-temperature 3D molecular crystals (Cr3D) to the hierarchical structure involving Coll. The smaller transitions are related to the coexisting lamellar structures.

A subtle question arises regarding what limits the nanoplatelet lateral growth to allow 2D-platelets with their well-defined colloidal length-scale self-assembly. A potential mechanism is suggested by rod–coil block polymers, where the lateral growth of the layers is controlled. Therein, the molecular rods (mesogens) can pack tightly side-by-side into layers, whereas the molecular coils (acting like “brushes” above and below the mesogen layers) involve larger lateral dimensions, thus leading to packing frustrations.^7,44^ Therefore, the coils have to stretch while keeping the mesogen tightly packed, causing a progressively increasing entropic penalty when increasing the number of molecules that are laterally added, thus limiting the colloidal plate lateral size. We suggest a similar mechanism in the present case (Figure 4a). The radius of gyration *R*_g_ of free alkyl chains of length *n* in bulk has been modeled to obey *R*_g_ = 1.69*n*^0.549^ (Å).^45^ Therefore, one can estimate *R*_g_ ∼ 7.2 Å for the tetradecyl chain, which is considerably smaller than the contour length, inferring coiling. The coiling can be observed by temperature-dependent Fourier-transform infrared spectroscopy (FTIR) measurements where, at 160 °C, the alkyl tails show the CH_2_ symmetric and asymmetric stretching vibrations at 2925 and 2854 cm^–1^, respectively, indicating *gauche*-conformation that corresponds with the molten state of the alkyl tails^46,47^ (Figure 5a). Thus, we suggest that the zwitterions ionically pack tightly in layered configurations due to their high electrostatic interactions, where the lateral colloidal plate sizes are controlled by an alkyl stretching penalty. The exact shape of the colloidal 2D sheets cannot be assessed at this stage. Early work by Narayan et al. has suggested disc-shaped aggregates upon heating sodium ricinoleate.^43^ The domain sizes of the colloidal assembly were evaluated using the Scherrer equation to be ca. 54 nm (see Table S5, which also discloses the approximative domain sizes of all phases).

The above characterizations suggest highly complex structures and polymorphism depending on the temperature. The observed coexistent lamellar phases Sm1, Sm2, and Sm3 all show different but equidistant reflections in the SAXS graphs (Supporting Information Section S4). Polymorphism has been observed in thermotropic LCs, wherein different types of packings are observed depending on the cooling rate and the thermal history.^22,48−58^ A detailed study of the coexisting/metastable phases was outside the scope of the current focus, but they could arise from kinetically trapped states that are unable to relax below the clearing point. These lamellar phases have different rigidities corresponding to the state of the alkyl tails, as seen by the temperature-dependent FTIR measurements (Figure 5a). At 160 °C, the alkyl tails show the CH_2_ symmetric and asymmetric stretching vibrations at 2925 and 2854 cm^–1^, respectively, corresponding to the *gauche*-conformation, which indicates that the alkyl tails are in the molten state. Upon cooling to 30 °C, these vibrations shift to lower frequencies (2919 and 2850 cm^–1^, respectively), which are characteristic of the *trans*-conformation typically observed in the crystalline state. It is likely that the coexistent lamellar phases could be due to undulations in the lamellar thicknesses due to packing frustrations and kinetic factors, as the phase transition dynamics are slowed down by the high viscosity phases.^59,60^

DSC provides additional information, wherein a large peak at 149–155 °C (heating) and 133 °C (cooling) is seen, connected with the transition from Cr3D to Coll, involving hysteresis (Figure 5b). The minor transitions at ca. 49 °C (cooling) and ca. 66 °C (heating) and the slope of the thermogram baseline in the heating scan can be assigned to the transitions of the metastable/coexisting states.

### 2.3 Plasticization of the Zwitterionic Domains to Relieve the Packing Frustration for the High-Temperature Colloidal Self-Assembly

**Figure 6:**
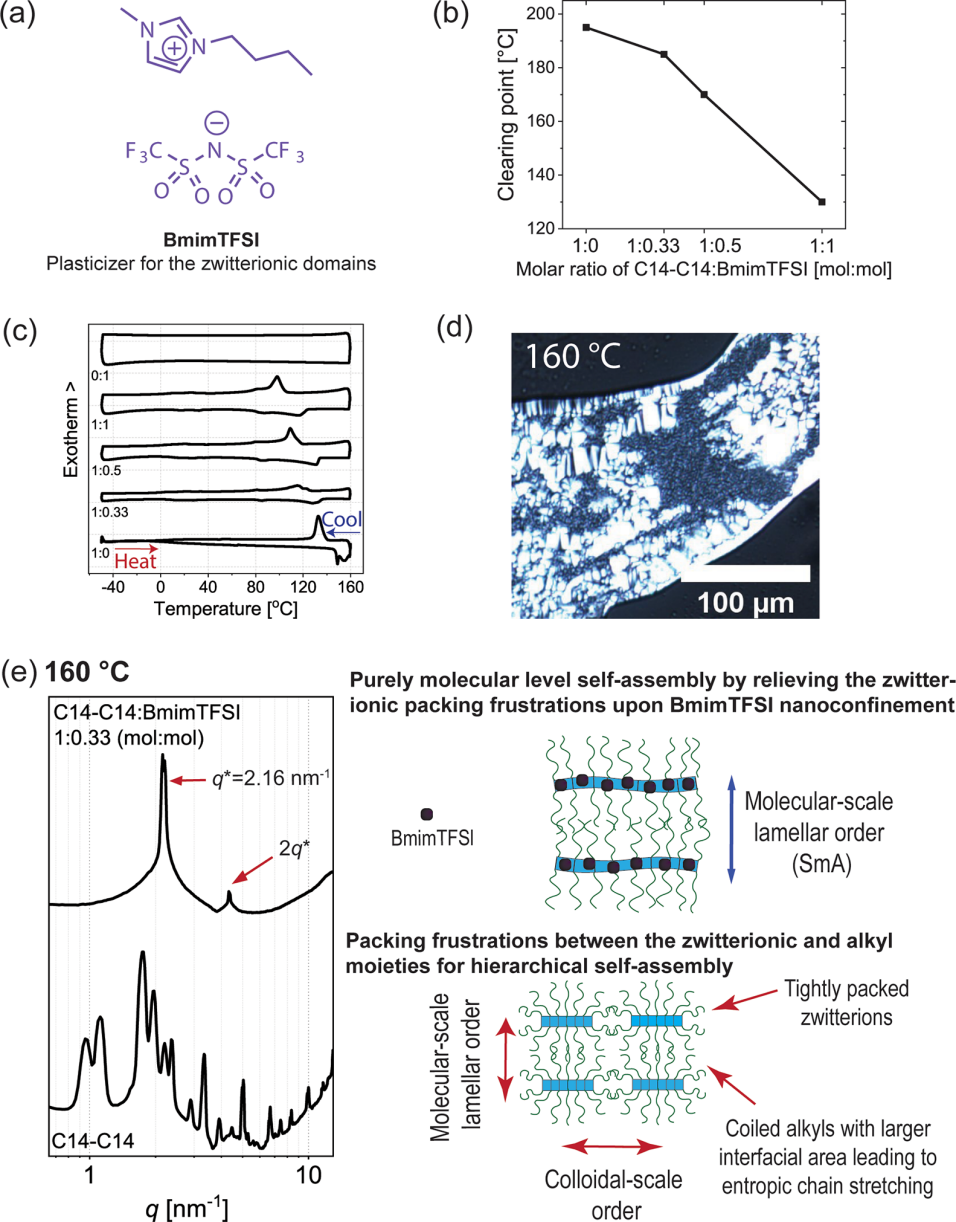
Plasticization of the electrostatic interactions in alkyl zwitterionic molecules by ionic liquids to relieve the packing frustration of the hierarchical self-assembly to allow smectic liquid crystallinity. (a) Chemical formula of BmimTFSI used as a plasticizer. (b) Clearing point upon adding BmimTFSI plasticizer to C_14_–C_14_ in quick thermal sweeps. (c) DSC showing the shift of the crystallization transition temperature upon adding different molar fractions of BmimTFSI to C_14_–C_14_. (d) POM of C_14_–C_14_/BmimTFSI 1:0.33 mol/mol at 160 °C, showing oily streaks and Maltese crosses, characteristic for smectic A liquid crystals. (e) X-ray data for C_14_–C_14_/BmimTFSI 1:0.33 mol/mol showing smectic LC unlike the unplasticized C_14_–C_14_, which shows complex self-assemblies involving colloidal and molecular length scales. This indirectly supports the notion that the packing frustrations of C_14_–C_14_ have been relieved.

Above, it was suggested that at high temperatures, hierarchical self-assemblies were achieved, also leading to complexity, as there is packing frustration between the tightly packed zwitterionic moieties and coiled alkyl chains. This could be indirectly proven by relieving the zwitterionic tight packing by their nanoconfined plasticization. Therefore, we next searched for a direct plasticization of the polar zwitterionic part using low-melting, highly polar, and nonvolatile compounds to screen the strong Coulombic interactions between the zwitterionic moieties. Therein, inspiration was found in the effects and screening of the ionic interactions by, e.g., tuning the ionic strength in aqueous media.^61−66^ Ionic screening has been previously used to control the lateral packing of aggregate structures in the mineral phase.^67^ Herein, we selected a common ionic liquid, 1-butyl-3-methylimidazolium bis(trifluoromethylsulfonyl)imide (BmimTFSI), see Figure 6a, as a model plasticizer. Unlike in the classic ionically conducting assemblies, we did not search for high conductivity but instead for concepts to control the zwitterionic interactions in the solid state. Using rapid temperature scans shows that BmimTFSI reduces the isotropization temperature from 194 °C of pure C_14_–C_14_ to 185, 170, and 130 °C for 1:0.33, 1:0.5, and 1:1 mol/mol of C_14_–C_14_/BmimTFSI, respectively (Figure 6b). DSC also shows that the crystallization transitions were systematically reduced upon the addition of BmimTFSI (Figure 6c). Limiting to small fractions of plasticizer, i.e., C_14_–C_14_/BmimTFSI 1:0.33 mol/mol, heating to 160 °C leads to oily streaks and Maltese crosses, thus suggesting a SmA mesophase (Figure 6d). In SAXS, reflection at *q* = 2.15 nm^–1^ and its second-order reflection at twice the *q*-value are observed, confirming that the purely smectic A phase, without crystallization peaks, is achieved with a periodicity of 2.92 nm (Figure 6e and Supporting Information Section S6). Thus, we show a concept to screen ionic interactions in a solvent-free state. The peak shape is Lorentzian, suggesting relatively short-range positional order.^68^ Even if not aimed at in this study, these complexes can reach ionic conductivity up to 0.6 mS cm^–1^ at 150 °C (Supporting Information Section S7).

## 3 Conclusions

In conclusion, we show colloidal and molecular-level hierarchical complex self-assembly of zwitterionic bis-*n*-tetradecylphosphobetaines upon heating by combining long-range electrostatic and smaller-scale nanosegregation provided by the alkyl tails. At room temperature, coexistent phases are observed with purely molecular lamellar motifs. Upon heating, a transition to hierarchical self-assembly is observed, where an additional order involving oblique 2D colloidal nanoplatelet forms. As the layers are weakly correlated, shear flow is observed. The new well-defined colloidal lateral length scale between the nanoplatelets arises from the packing frustration between the tightly packed zwitterions and fluid alkyl chains. This is indirectly evidenced by relieving the lateral packing frustration upon plasticizing with common ionic liquid 1-butyl-3-methylimidazolium bis(trifluoromethylsulfonyl)imide, leading to the smectic-A mesophase at elevated temperatures. Plasticization by ionic liquids can be used as a general scheme to screen the strong electrostatic interactions in the solvent-free state, analogous to tuning ionic strength in the aqueous state. We foresee that different zwitterionic parts and functional ionic liquids can be selected, indicating the potential for a platform of modular functional self-assembled materials and new avenues toward 2D ionic crystalline materials.

## 4 Methods

Detailed description of methods and analyses is disclosed in the Supporting Information.

### 4.1 Synthesis of Alkyl Zwitterionic Molecules

Both symmetric and asymmetric bis-*n*-alkylphosphobetaines C_*m*_H_2*m*+1_–O–(PO^–^)–O–C_2_H_4_–N^+^(CH_3_)_2_–C_*n*_H_2*n*+1_ (denoted as C_*m*_–C_*n*_) were synthesized with alkyl tail lengths of C_6_–C_6_, C_8_–C_8_, C_10_–C_10_, C_12_–C_12_, C_14_–C_14_, C_8_–C_14_, and C_18_–C_8_. The synthesis was performed according to a previously published route. Details are in Supporting Information Section S1.^39,40^

### 4.2 Nuclear Magnetic Resonance

The synthesized compounds were characterized by ^1^H and ^13^C NMR with a Bruker AV III 400 spectrometer in CDCl_3_.

### 4.3 Fourier Transform Infrared Spectroscopy

FTIR was performed by using a Thermo Nicolet 380 FT-IR spectrometer. Temperature-varied FTIR measurements were performed with the spectrometer equipped with a Specac Electrical Heating Jacket and a solid sample holder. The sample was measured in a KBr pellet in the transmittance mode.

### 4.4 Thermogravimetric Analysis

TGA was performed by using a TA Instruments TGA Q500 instrument at 40–900 °C with a scanning rate of 10 °C/min under N_2_.

### 4.5 Karl Fischer Titration

The adsorbed water content was determined by indirect coulometric Karl Fischer titration using the heating method, where the adsorbed water was released by heating the sample to 150 °C.

### 4.6 Preparation of C_14_–C_14_/BmimTFSI (mol/mol) Complexes

A known mass of the C_14_–C_14_ was weighed in a vial, after which a known amount of a solution of 1-butyl-3-methylimidazolium bis(trifluoromethylsulfonyl)imide (BmimTFSI, 99.5%, Solvionic) in chloroform was added to yield the targeted molar ratios. The mixture was heated slightly and mixed to fully dissolve the zwitterion in the solvent. The chloroform was evaporated out in a fume hood, leaving the slowly assembled mixture in a vial. Prior to measurements, the dried mixtures were heated to 160 °C to ensure full mixing of the components.

### 4.7 Differential Scanning Calorimetry

DSC thermograms of the samples were measured by using a TA Instruments MT-DSC Q2000 instrument. Runs were performed under a N_2_ atmosphere. A known amount of sample (4–6 mg) was measured in an aluminum pan, which was first cooled to −50 °C at 10 °C min^–1^, equilibrated for 2 min. Then, at least two heating–cooling cycles were performed between −50 and +160 °C with a scanning rate of 10 °C min^–1^. The second heat–cool cycle was used in the analyses.

### 4.8 X-ray Characterization

Temperature-dependent SAXS/WAXS measurements were carried out at the European Synchrotron Radiation Facility (ESRF) in Grenoble, France, at the (French) CRG beamline BM02-D2AM. The measurements were performed with an incident photon energy of 16 keV, corresponding to a wavelength of 0.07749 nm. The samples were dried, degassed, and sealed in closed glass capillaries. The utilized temperature range was 25 to 160 °C, with a heating/cooling rate of 1 °C min^–1^. Scans were recorded every 5 °C/10 °C (cooling/heating) after an equilibration time of 2 min at the given temperature set points. An empty capillary was used as a background. The SAXS 1D patterns were first fitted with Scatter,^69^ using the built-in lamellae model. VESTA^70^ software was used to analyze the crystallographic parameters further. Additional X-ray measurements were performed on XENOCS Xeuss 3.0, where the samples were measured in open glass capillaries on a temperature-controlled heat plate.

### 4.9 Polarized Optical Microscopy

The thermal behavior of samples was characterized using a Leica DM4500 polarizing optical microscope with ×5, ×10, ×20, and ×40 objectives, a Linkam LTS350 heating stage, a CI94 temperature controller, and a LN2 cooling system. A small amount of the sample was placed atop a glass microscope slide and covered with a thin cover glass. POM analyses were performed using three heating–cooling cycles: first, a heating–cooling cycle to allow the mixing of components and the potential kinetically trapped states to transition to their thermodynamic equivalents. The thermotropic behavior was studied during the second heating–cooling cycle, after which the samples were allowed to stand at room temperature to study the metastable phases.

### 4.10 Electrochemical Impedance Spectroscopy

Ionic transport properties were studied using a HF2IS impedance spectroscope (Zurich Instruments). The samples were measured in MicruX ED-IDE1-Au-interdigitated electrodes (averaged cell constant 0.0276 cm^–1^ from the manufacturer) that were glued onto microscope slides with copper wires directly soldered onto the electrode cells. A small amount of sample was placed on the electrode, and the sample was heated up to its flowing state to fill the cell. The filled sample cell was dried in a vacuum oven overnight at 25–40 °C prior to measurement. At the start of the measurement, the cell was heated to 150 °C and stabilized for 20 min. The ionic transport measurements were then performed during cooling scans from 150 to 0 °C at a rate of 1 °C min^–1^, and the equilibration time was 3 min at the measurement temperature to ensure a thermal equilibrium. A Linkam LTS350 heating plate, a CI94 temperature controller, and a LN2 cooling system were used. In the EIS measurement, a 4-terminal measurement setup with an AC excitation of 100 mV was applied, and the impedance spectroscopy was performed over the 10 MHz to 10 Hz frequency range. The analysis of EIS data was performed by using ZView software (Scribner Associates). Additional information on the EIS can be found in Supporting Information Section S7.

## Supporting Information Available

Synthesis and characterization of bis-*n*-alkylphosphobetaines (C_*m*_–C_*n*_), thermal stability of bis-*n*-alkylphosphobetaines (C_*m*_–C_*n*_), POM studies of bis-*n*-alkylphosphobetaines (C_*m*_–C_*n*_), structural characterization of C_14_–C_14_, the metastable/coexisting phases, phase behavior of C_14_–C_14_:BmimTFSI mixtures, and EIS (PDF)
