# Supplementary material for: Heating-Induced Switching to Hierarchical Liquid Crystallinity Combining Colloidal and Molecular Order in Zwitterionic Molecules
Source: ACS Omega. 2023 Oct 12;8(42):39345–53. doi: 10.1021/acsomega.3c04914 (PMC10601052; doi:10.1021/acsomega.3c04914)
Supplement: Supplementary file 1 — ao3c04914_si_001.pdf [file ao3c04914_si_001.pdf]

Supporting Information

Heating-Induced Switching to Hierarchical Liquid Crystallinity Combining Colloidal and Molecular Order in Zwitterionic Molecules

Lotta Gustavsson[a], Zhong-Peng Lv[a]*, Tomy Cherian[a], Wille Seppälä[a], Ville Liljeström[b], Bo Peng[a]*, Simo Huotari[c], Patrice Rannou[d], Olli Ikkala[a]*

[a] *Department of Applied Physics, Aalto University, Puumiehenkuja 2, FI-00076, Espoo, Finland*

[b] *Nanomicroscopy Center, Aalto University, Puumiehenkuja 2, FI-00076, Espoo, Finland*

[c] *Department of Physics, University of Helsinki, P.O. Box 64, FI-00014, Helsinki, Finland*

[d] *Université Grenoble Alpes, Université Savoie Mont-Blanc, CNRS, Grenoble INP, LEPMI, 38000 Grenoble, France*

*Email: zhongpeng.lyu@aalto.fi
*Email: bo.peng@aalto.fi
*Email: olli.ikkala@aalto.fi

Table of Contents

Supporting Section 1. Synthesis and characterization of bis-*n*-alkylphosphobetaines (C_m-C_n)

Supporting Section 2. Thermal stability of bis-*n*-alkylphosphobetaines (C_m-C_n)

Supporting Section 3. Polarized optical microscopy studies of bis-*n*-alkylphosphobetaines (C_m-C_n)

Supporting Section 4. Structural characterization of C_{14}-C_{14}

Supporting Section 5. The metastable/coexisting phases

Supporting Section 6. Phase behavior of C_{14}-C_{14}:BmimTFSI mixtures

Supporting Section 7. Electrochemical Impedance Spectroscopy

References

Supporting Section 1: Synthesis and characterization of bis-*n*-alkylphosphobetaines (C_m-C_n)

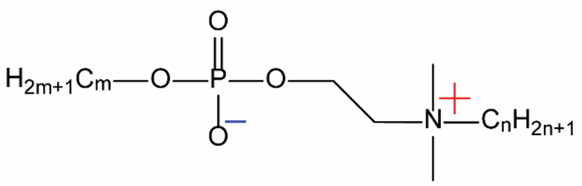

Zwitterionic amphiphiles Cm-Cn	
Symmetric	C_6-C_6
	C_8-C_8
	C_{10}-C_{10}
	C_{12}-C_{12}
	C_{14}-C_{14}
Asymmetric	C_{18}-C_8
	C_8-C_{14}

Figure S1. Synthesized zwitterionic molecules.

A selected series of zwitterionic bis-*n*-alkylphosphobetaines C_mH_{2m+1}–O–(PO^-)–O–C_2H_4–$N^+(CH_3)_2$–C_nH_{2n+1} (denoted as C_m-C_n) were synthesized. The synthesis was performed according to a published route where phosphobetaine zwitterionic amphiphiles with alkyl tail lengths from C_4 to C_{22} have been obtained[1,2]. The yields corresponded with the previous synthesis protocols and were of ca. 13-30 %. All synthesized compounds were characterized using ^{1}H & ^{13}C NMR, and FTIR. The molecules are hygroscopic and were previously shown to crystallize as hydrates.[1,2] The amount of adsorbed water was determined by Karl Fischer titration as 6 wt% after storage in ambient conditions, and this adsorbed water can be removed during the typical drying procedure of heating the sample to 60 °C in vacuum overnight.

Characterization of bis-*n*-tetradecylphosphobetaine (C_{14}-C_{14})

^{1}H NMR (Bruker AV III 400 MHz, $CDCl_3$, 25 ºC, TMS): δ = 0.87 (t, *J*(H,H)=6.6 Hz, 3H), 1.24 (br m, 44H), 1.58 (tt, *J*(H,H)=6.9 Hz, 2H), 1.70 (br m, 2H), 3.35 (br s, 6H), 3.47 (br m, *J*(H,H)=7.4 Hz, 2H), 3.82 (br m, *J*(H,H)=6.4 Hz, 4H), 4.29 (br m, 2H) ppm

^{13}C NMR (Bruker AV III 100 MHz, $CDCl_3$, 25 ºC, TMS): δ = 14.07, 22.65, 22.85, 25.90, 26.35, 29.33, 29.44, 29.51, 29.64, 29.70, 31.05, 31.89, 51.71, 55.87, 63.99, 65.50 ppm

FTIR (Thermo Nicolet 380): ν = 2959-2850 (vs, C-H stretch), 1466 (s, CH_2 deformation), 1242 (vs, P=O stretch), 1076-1100 (s, C-O stretch), 1055 (s, P-O-C stretch) cm^{-1}

MS (QTOF-MS) MH^+=562.49 (Theoretical M=561.49 g/mol)

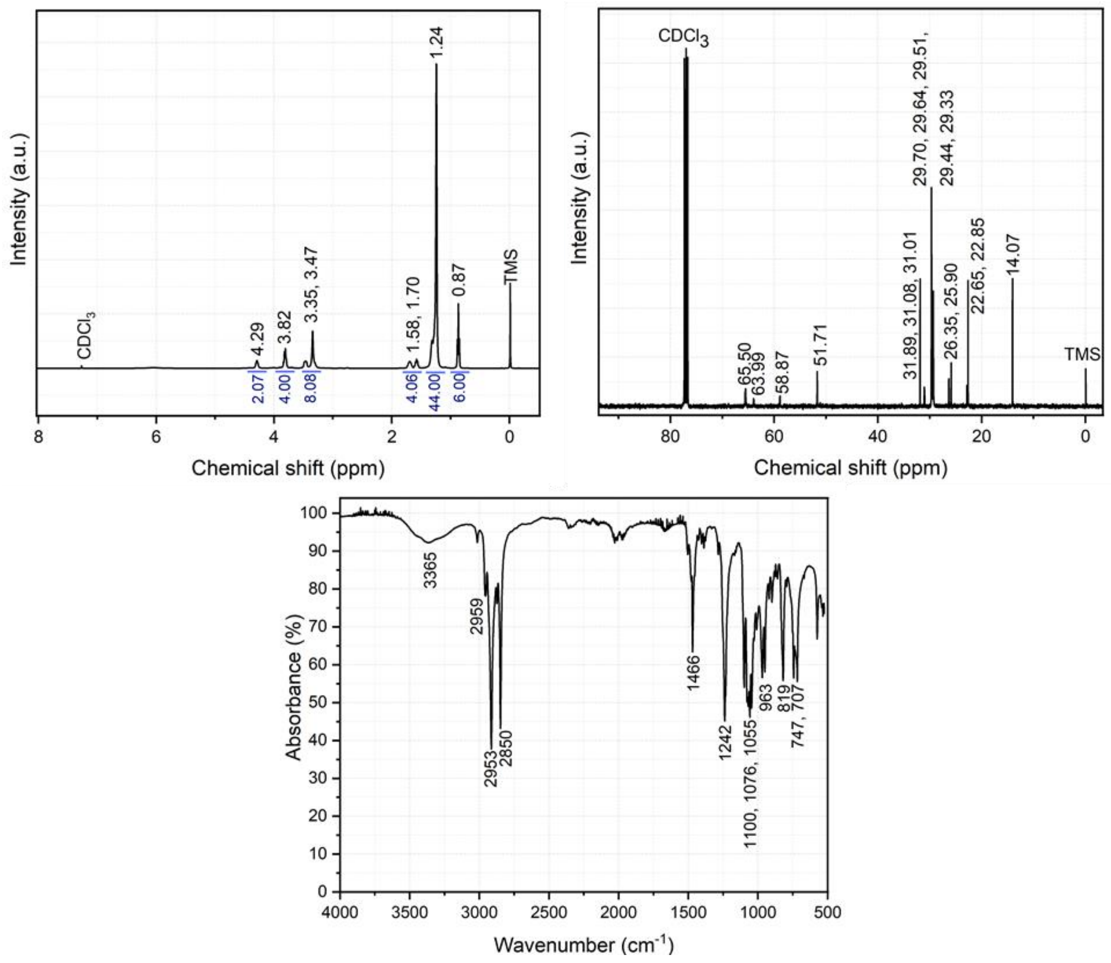

Figure S2. ^{1}H & ^{13}C NMR, and FTIR characterizations of C_{14}-C_{14}.

Supporting Section 2: Thermal stability of bis-*n*-alkylphosphobetaines (C_m-C_n)

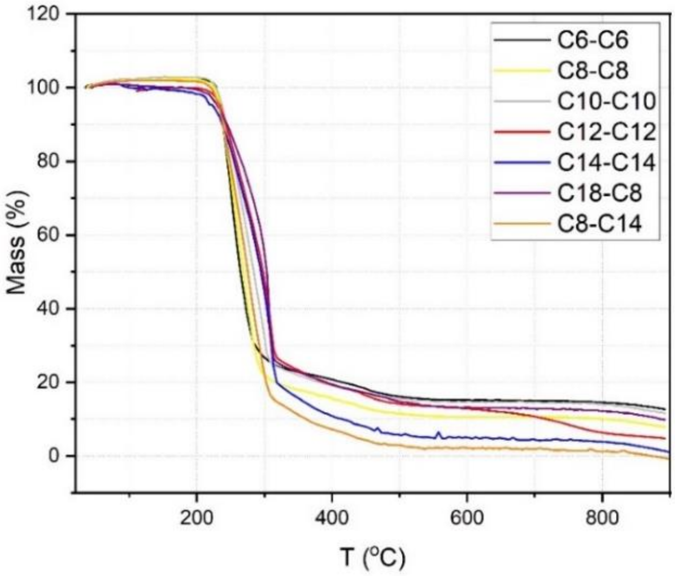

Figure S3. The thermal stability of the compounds was studied with thermogravimetric analysis (TA Instruments TGA Q500) at 40-900 °C under N_2. The TGA-determined decomposition temperature, marking the decomposition of the *n*-alkyl chains starts at slightly above 200 °C for all compounds. However, quaternary ammoniums are known to show melting accompanied with chemical degradation. This decomposition can occur in at least two mechanisms: reverse Menschutkin reaction or Hoffman elimination,[3] both of which conserve the alkyl tails, leading to non-volatile decomposition products which are not seen in the TGA thermogram. The thermostabilities were additionally studied by SMP30 melting point apparatus (Stuart) with visual inspection. The zwitterionic bis-*n*-alkylphosphobetaine molecules showed i) a transition to a visually gel-like state, followed by ii) a coloration process starting at ca. 180-190 °C.

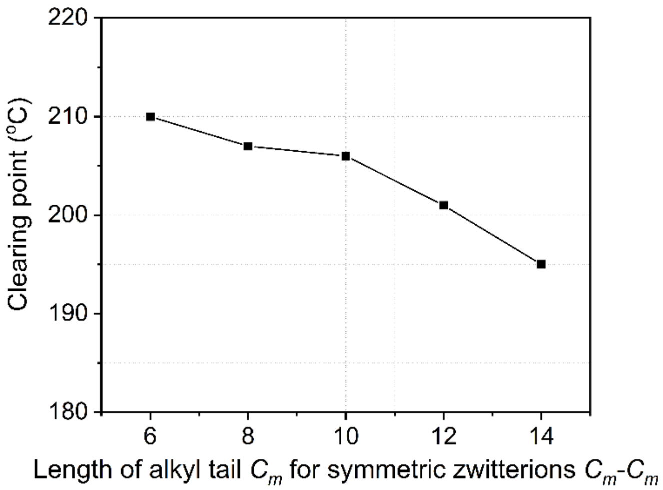

Figure S4. Clearing points for synthesized symmetric zwitterionic phosphobetaine compounds as a function of their alkyl tail lengths under rapid thermal scans.

Supporting Section S3: Polarized optical microscopy studies of bis-*n*-alkylphosphobetaines (C_m-C_n)

The series of bis-*n*-alkylphosphobetaine molecules were studied until the isotropization through crossing their respective clearing temperature (T_{iso}) to investigate their full thermal behavior. In the optical microscope studies, all synthesized compounds behaved in a similar manner with slightly shifted transition temperatures.

All samples showed a gradual increase in birefringence, a gradual softening of the crystals, and a short-lived smectic mesophase, where the typical oily streak and Maltese cross textures were observed right before the isotropization. The smectic textures were visible for longer time the longer the *n*-alkyl tails, owing to the increased amphiphilic nature. As expected, the clearing temperature was lower for the longer *n*-alkyl tail: T_{iso} was 195 °C for the C_{14}-C_{14} (longest tail length) and 210 °C for C_6-C_6 (shortest tail length). Upon cooling from the isotropic state, the LC textures (bâtonnet, focal conic fan, and Maltese crosses) of bis-*n*-alkylphosphobetaine molecules were fully developed and unambiguously identified.

When heated to 160 °C (fixed as a safe temperature of investigation, *vide supra*), no characteristic LC textures could be observed, as seen by the POM microphotograph in the main text. The POM studies of both temperature ranges (i.e., below 160°C and till reaching the isotropic state) were therefore instrumental in understanding the complex thermal behavior of the bis-*n*-alkylphosphobetaine molecules.

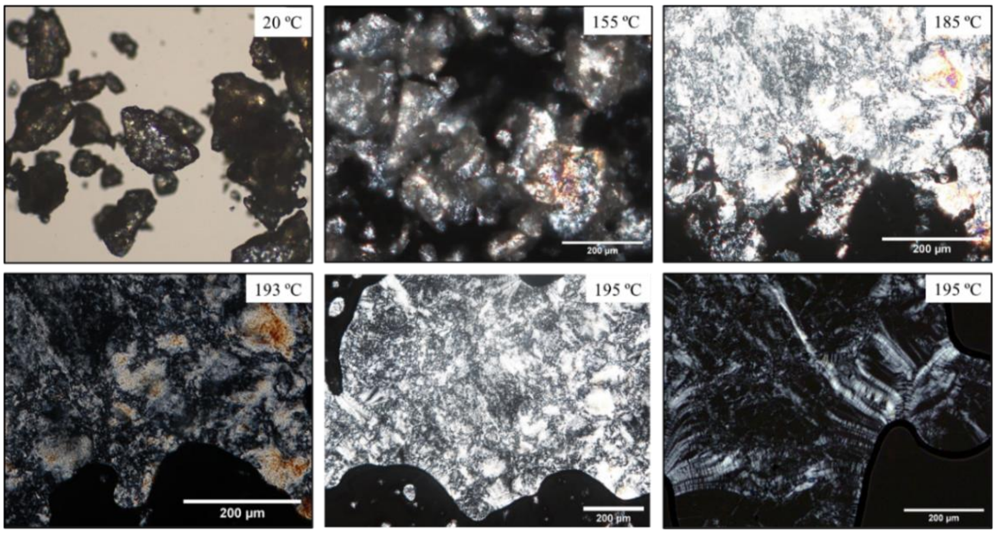

Figure S5. Selected POM microphotographs for C_{14}-C_{14} during the 1st heating scan. The crystals show continuously increasing birefringence with heating. While the birefringence increases, the crystals also become gradually softer. The fluidity increases continuously, and by 180 °C the sample is spontaneously (in the absence of any mechanical solicitation like pressing or shearing) flowing though being viscous. It then starts to show the characteristic birefringent oily streak textures for a smectic liquid crystal at 195 °C that disappear on further stay on that temperature as the sample transitions into the isotropic state across its clarification temperature. The scale bars are 200 µm.

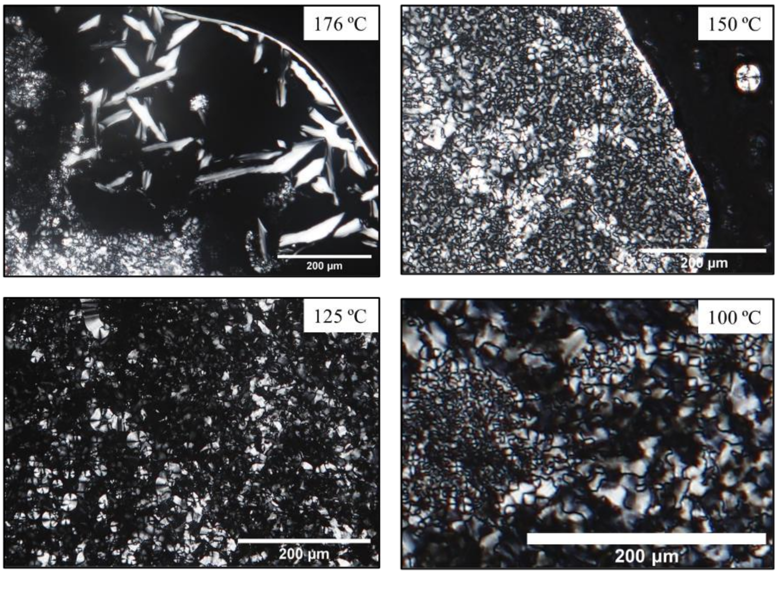

Figure S6. Selected POM microphotographs for C_{14}-C_{14} on cooling scan from the isotropic state. Typical birefringent LC textures are observed: bâtonnets that grow into focal-conic fans, Maltese crosses (clean and distorted), and finally paramorphotic mosaic textures, the latter ones being indicative of ordered smectic phases (e.g. SmB_{cryst} or SmE) developing as a result of increasing intra- vs interlamellar correlations. The high viscosity of the liquid crystalline phases led to the freezing of the textures when decreasing temperature towards room temperature. The scale bars are 200 µm.

Supporting Section S4: Structural characterization of C_{14}-C_{14}

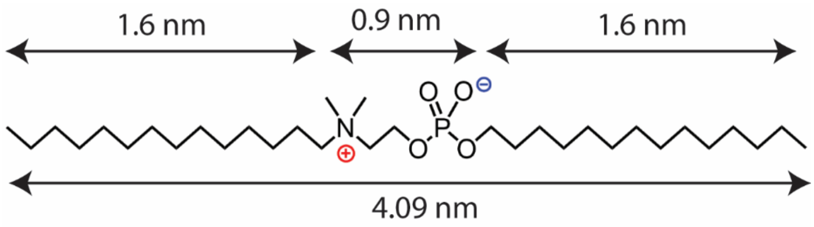

Figure S7. Characteristic dimensions of C_{14}-C_{14}.

The structures of C_{14}-C_{14} are listed for three selected temperatures: 25, 55, and 160 ºC, in the following tables. At room temperature, an orthorhombic model was fitted for Cr3D but with increased temperature, the fitting starts to deviate from ideality. We propose a gradual tilting of the crystallographic axis of the Cr3D structure, i.e., from orthorhombic to monoclinic, and ultimately to triclinic geometries. Due to the complexity of the triclinic structure, the lattice parameters (a, b, c, α, β, γ) are not explicitly stated.

In the tables, the following abbreviations are used:

q_{exp} experimentally observed peak position as a scattering vector q

q_{theor} theoretical peak position as scattering vector q, as calculated using the lattice parameters $a, b, c, \alpha, \beta, \gamma$

Sm_2			Cr3D		
Lamellar<br>a = 4.45 nm			*Orthorhombic*<br>a = 0.93 nm, b = 0.88 nm,<br>c = 3.96 nm		
(hkl)	q_{exp} (nm^{-1})	q_{theor} (nm^{-1})	*(hkl)*	q_{exp} (nm^{-1})	q_{theor} (nm^{-1})
(001)	1.43	1.41	(001)	1.59	1.59
(002)	2.83	2.82	(002)	3.18	3.17
(003)	4.25	4.23	(003)	4.77	4.76
(004)	5.62	5.64	(004)	6.35	6.34
(005)		7.06	(005)	7.93	7.93
(006)	8.56	8.47	(006)	9.51	9.51
			(007)	11.09	11.10
			(008)	12.69	12.69
			(100)	6.79	6.79
			(010)	7.14	7.14
			(012)	7.83	7.81
			(110)	9.82	9.85
			(112)	10.47	10.35
			(1$\bar{1}$2)		
			(116)	13.67	13.70
			(203)	14.45	14.39
			(023)	14.96	15.05
			(211)	15.45	15.42
			ion	13.0	

Table S1. X-ray reflections of C_{14}-C_{14} at 25 °C.

Sm_2			Sm_1			Cr3D		
Lamellar<br>a = 4.35 nm			*Lamellar*<br>a = 3.61 nm			*Monoclinic*<br>a = 0.93 nm, b = 0.88 nm,<br>c = 3.97 nm, γ = 88.5°		
(hkl)	q_{exp} (nm^{-1})	q_{theor} (nm^{-1})	*(hkl)*	q_{exp} (nm^{-1})	q_{theor} (nm^{-1})	*(hkl)*	q_{exp} (nm^{-1})	q_{theor} (nm^{-1})
(001)	1.46	1.44	(001)	1.75	1.74	(001)	1.59	1.58
(002)	2.88	2.89	(002)	3.48	3.48	(002)	3.17	3.17
(003)	4.33	4.33	(003)		5.22	(003)	4.76	4.75
(004)	5.73	5.77				(004)	6.34	6.34
(005)		7.22				(005)	7.92	7.92
(006)	8.62	8.66				(006)	9.49	9.50
						(007)	11.07	11.09
						(008)	12.67	12.67
						(100)	6.68	6.74
						(010)	7.04	7.1
						(012)	7.72	7.78
						(110)	9.70	9.66
						(112)	10.28	10.17
						$(1\bar{1}2)$	10.51	10.41
						(116)	13.60	13.55
						(203)	14.27	14.29
						(023)	14.96	14.98
						(211)	15.22	15.15
						ion	13.02	

Table S2. X-ray reflections of C_{14}-C_{14} at 55 ºC.

Cr2D					
Lamellar<br>c = 3.81/3.76 nm			*Oblique (2D)*<br>a = 7.15 nm, b = 6.17 nm, γ = 114.1°		
(hkl)	q_{exp} (nm^{-1})	q_{theor} (nm^{-1})	*(hkl)*	q_{exp} (nm^{-1})	q_{theor} (nm^{-1})
(001)	1.55 (shoulder)	1.65	(100)	0.962	0.962
		1.67	(010)	1.12	1.12
(002)		3.30	(110)	1.74	1.74
	3.34	3.34	(200)		1.92
(003)	4.98	4.94	(020)	2.20	2.23
	5.04	5.01	(300)	2.90	2.89
(004)	6.61	6.59	(040)	4.47	4.46
	6.68	6.68	(330)	5.28	5.23
(005)	8.25	8.24	(050)	5.58	5.58
	8.36	8.35	(060)	6.68	6.69
(006)	9.87	9.89	ion	12.93	
	10.00	10.00			
(007)	11.52	11.54			
	11.67	11.68			

Table S3. The 2D crystal X-ray reflections of C_{14}-C_{14} at 160 °C.

Sm_3			Sm_1		
Lamellar<br>a = 2.67 nm			*Lamellar*<br>a = 3.21 nm		
(hkl)	q_{exp} (nm^{-1})	q_{theor} (nm^{-1})	*(hkl)*	q_{exp} (nm^{-1})	q_{theor} (nm^{-1})
(001)	2.37	2.35	(001)	1.96	1.96
(002)	4.76	4.70	(002)	3.91	3.91
(003)	7.05	7.05	(003)	5.79	5.87

Table S4. X-ray reflections of C_{14}-C_{14} at 160 °C, in the smectic polymorphs.

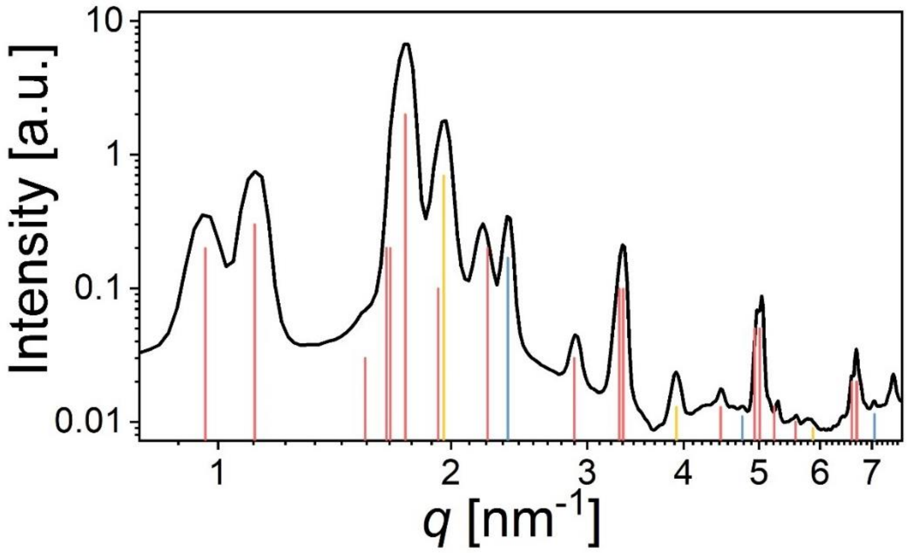

Figure S8. A close-up of the 1D SAXS pattern of C_{14}-C_{14} at 160 °C, showing the faint 2nd and 3rd order peaks of the Sm_3 structure (blue) along with the other structures, namely Sm_1 (yellow) and Cr2D (red).

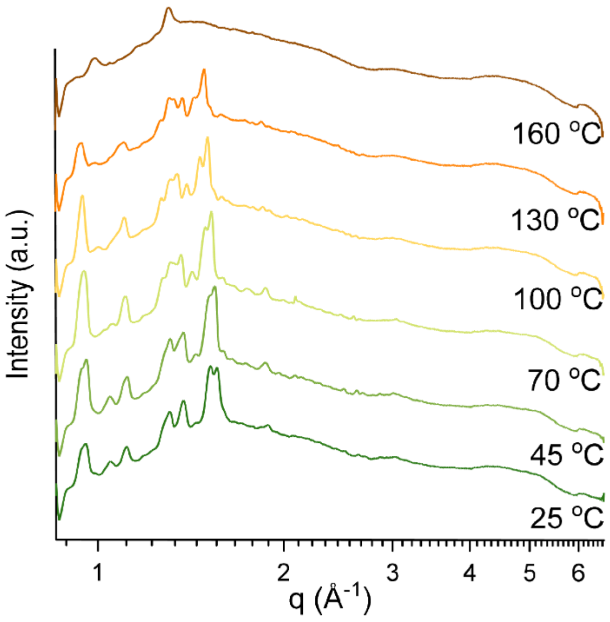

Figure S9. WAXS 1D profiles for C_{14}-C_{14} at selected temperatures during a cooling scan. The WAXS 1D profile recorded at 160 °C indicates the loss of molecular crystallinity. The changes in peak pattern and intensity observed upon cooling are due to the slow crystallization of the alkyl chains.

**Temperature**	**Peak identity**		**Δq_{fwhm} (nm^{-1})**	**Domain size (nm)**
**160 °C**	Cr2D	Oblique 2D (300)	0.105	54
		Lamellar (002)	0.0937	60
	Sm_1 (002)		0.120	47
	Sm_3 (001)		0.079	72
**130 °C**	Cr3D (002)		0.070	81
	Sm_1 (002)		0.128	44
**55 °C**	Sm_2 (002)		0.112	50
	Cr3D (002)		0.078	72
	Sm_1 (002)		0.231	24
**25 °C**	Sm_2 (002)		0.109	52
	Cr3D (002)		0.0711	79

Table S5. The domain sizes evaluated by the Scherrer equation*. In the analysis, data extracted from cooling scan were used to calculate the domain sizes for the structures. The (002) Bragg peaks were used instead of the (001) ones because the deconvolution could bring unnecessary error in the calculations. The instrumental line broadening is not taken into account in these calculations, and thus, while the presented domain size values cannot be considered as absolute values, they show qualitatively the size differences of the different phases.

*The domain sizes of phases can be calculated using the Scherrer equation:

$\tau = \frac{2\pi K}{\Delta q_{FWHM}} = \frac{5.65}{\Delta q_{FWHM}}$, where:

τ = mean crystallite domain size
K = dimensionless shape factor , commonly 0.9 < K < 1.0, here 0.9
Δq_{FWHM} = line broadening (full width at half maximum, FWHM)

Supporting Section 5: The metastable/coexisting phases

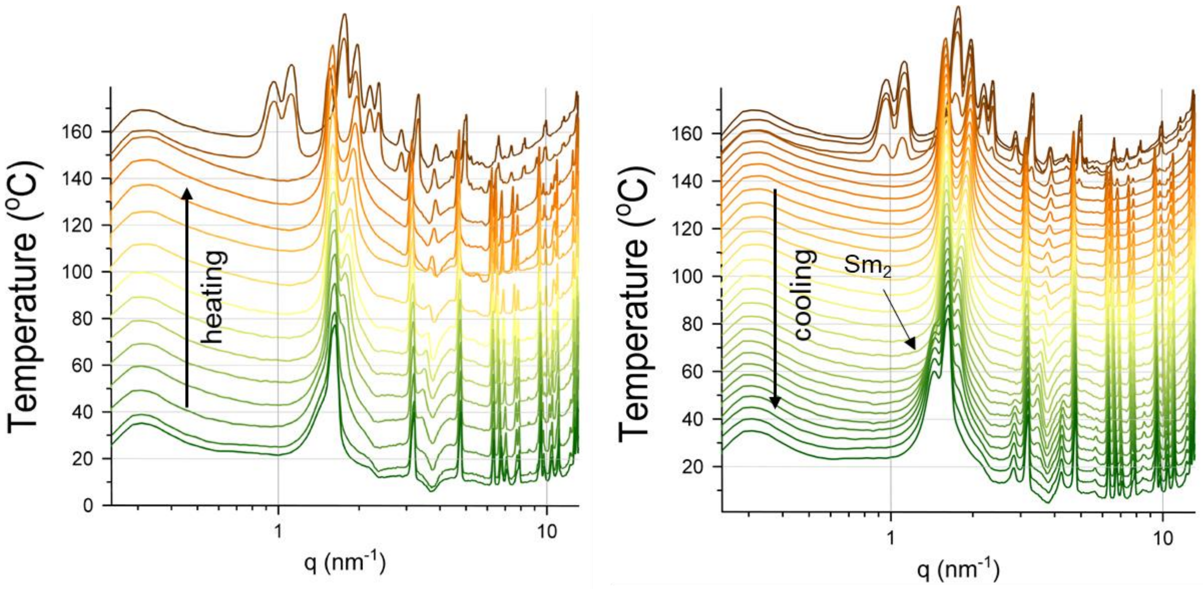

Figure S10. Temperature-dependent small angle X-ray scattering (SAXS) 1D profiles of C_{14}-C_{14} recorded during heating (left) and cooling (right) scans. The pointed Sm_2 phase is monotropic and seen only on the cooling scan. The coexisting phases could arise from kinetically trapped states that are unable to relax below the clearing point, but they were not studied further here.

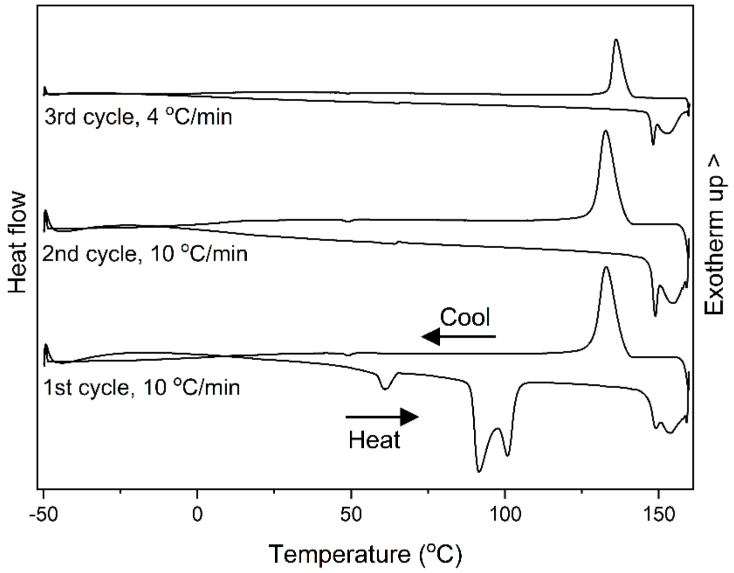

Figure S11. The DSC thermograms of C_{14}-C_{14} during the first three heating cycles suggest that after the 1st heating cycle, the thermal transition between Cr3D and Coll phases are reversible. The 1st heating scan is different than the subsequent ones which is typical for ionic amphiphiles.

Supporting Section 6: Phase behavior of C_{14}-C_{14}:BmimTFSI mixtures

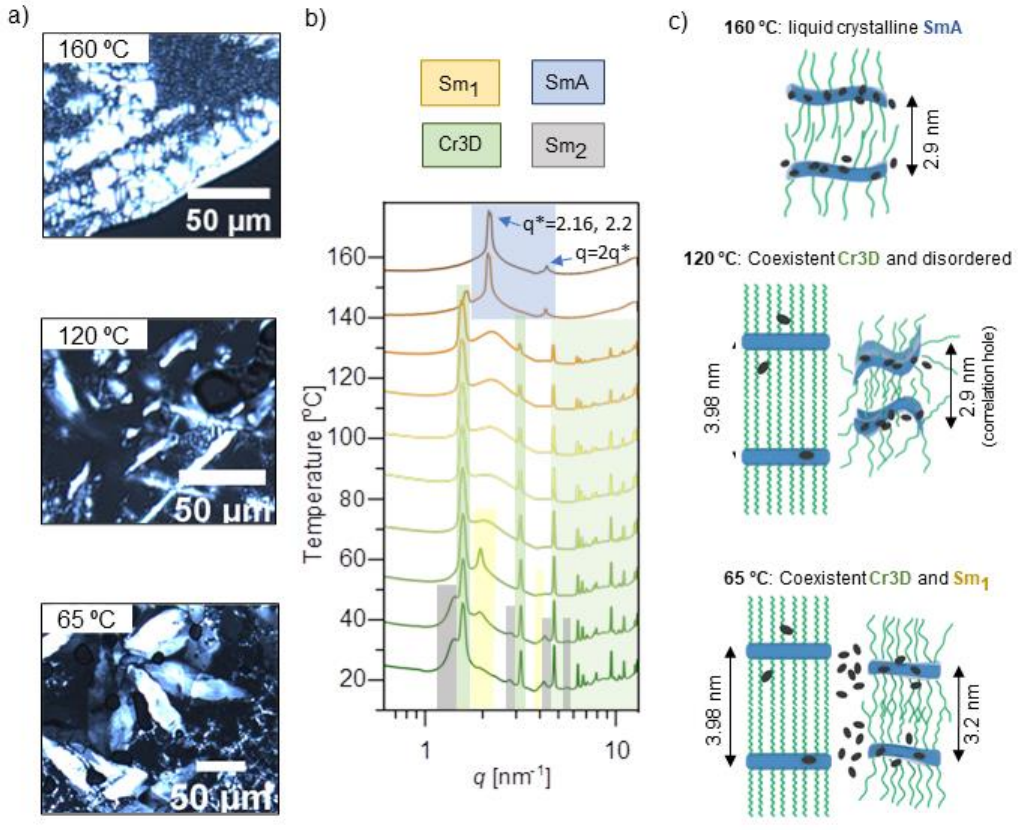

Figure S12. Phase behavior of C_{14}-C_{14}:BmimTFSI 1:0.33 mol:mol. a) POM microphotographs at selected temperature points. b) SAXS graphs grouped and color-coded. c) Schematic illustrations of structures at selected temperature points.

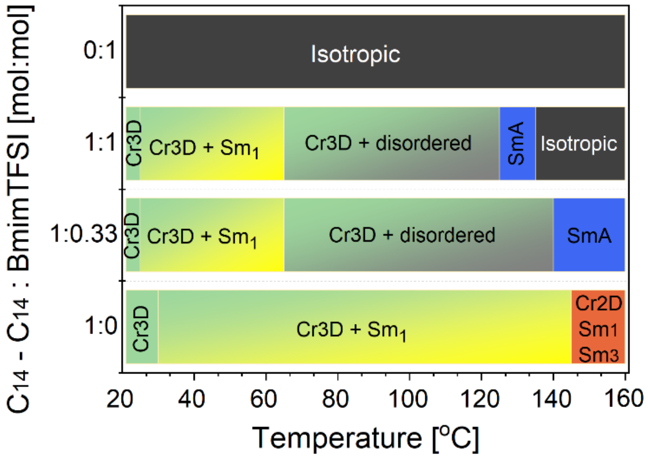

Figure S13. Phase chart for C_{14}-C_{14}:BmimTFSI mixtures.

Supporting Section 7: Electrochemical Impedance Spectroscopy (EIS)

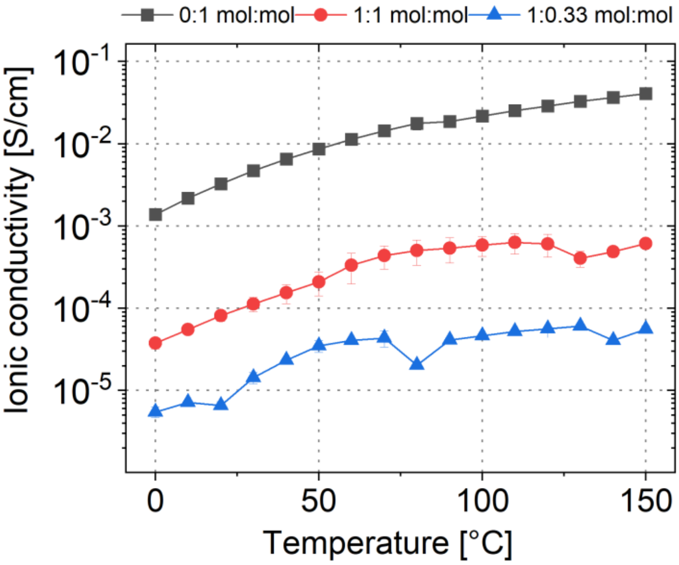

Figure S14. Ionic conductivity as a function of temperature for mixtures C_{14}-C_{14}:BmimTFSI 1:1 and 1:0.33 mol:mol. Referencing was performed by measuring a similar protocol of EIS measurements of BmimTFSI (0:1) throughout the temperature regime. The referencing were performed in triplicates. The calibration of the set-up and determination of systemic impedance was performed for each temperature point separately (every 10 ºC, over the temperature range of 0 to 150 ºC). The ionic conductivity values of BmimTFSI were calculated according to the Vogel-Tamman-Fulcher equation that correspond relatively well with limitedly available experimental data.[4,5]

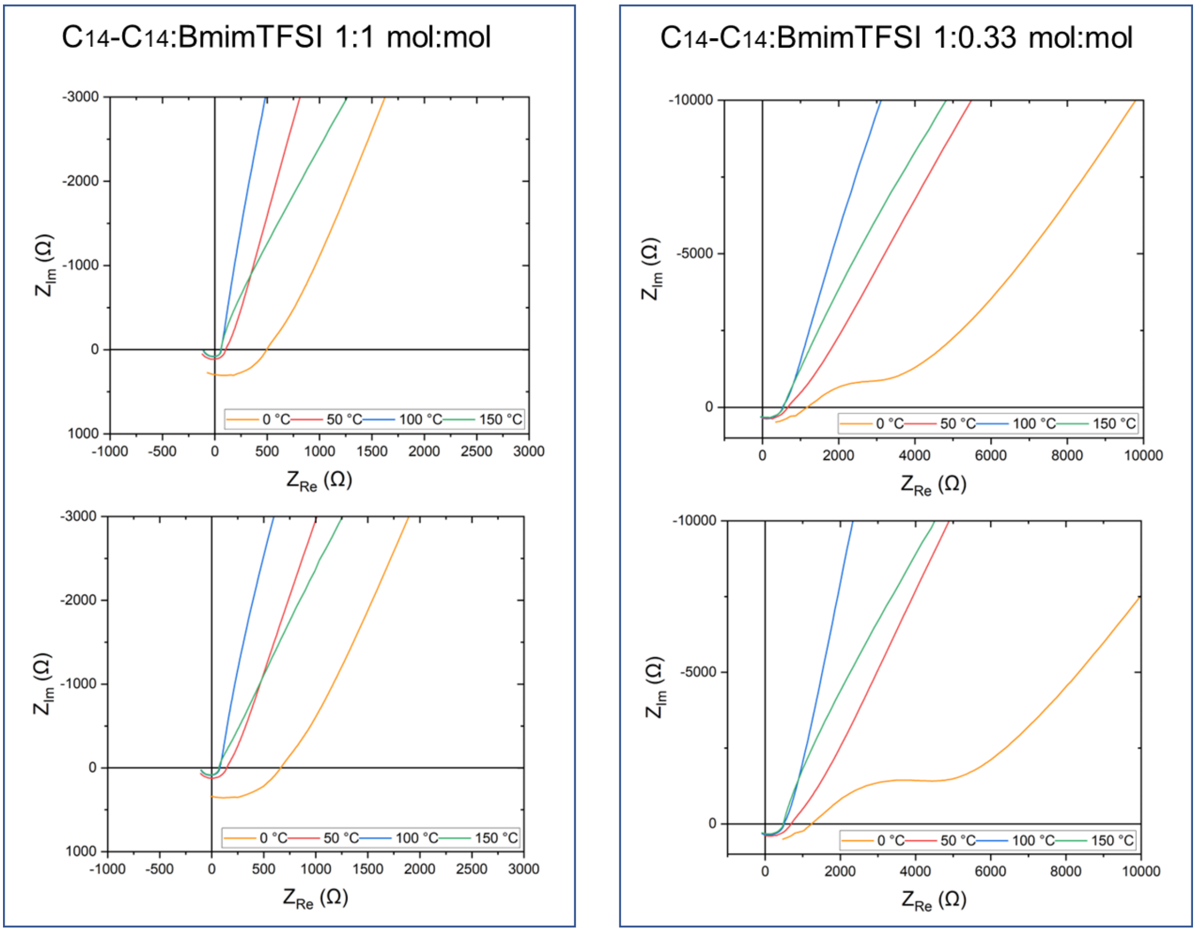

Figure S15. The Nyquist plots of the two replicate measurements of C_{14}-C_{14}:BmimTFSI 1:1 (left panel) and 1:0.33 (right panel) mol:mol at selected temperature points. The equimolar sample shows diffusive phenomenon throughout the temperature scan area 0-150 °C whereas for 1:0.33 mol:mol, a depressed capacitive semicircle develops in the low temperature regime 0-50 °C. The Nyquist plots were analyzed using ZView software, using the data analysis models shown in Figure S16.

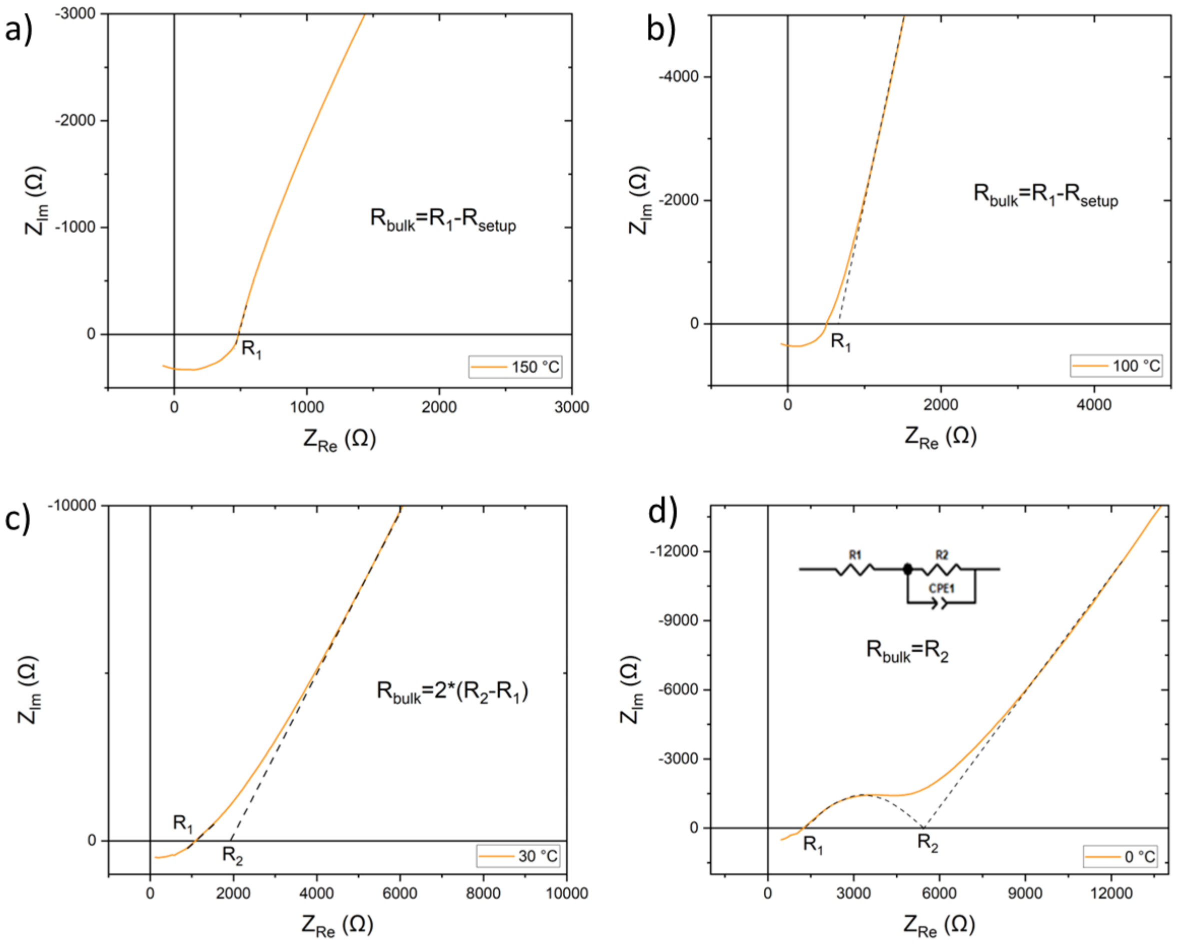

Figure S16. Schematic pictures showing the applied data analysis methods to ensure a proper fitting of the EIS data while relying on physics-based realistic and relevant models. The ionic conductivities were calculated using the formula $\sigma = \frac{K}{R_{bulk}}$, where K is the cell constant and R_{bulk} the bulk resistance extracted from the modelled EIS data. a) The straight line indicates a diffusion-controlled ion-conduction mechanism. b) Capacitive phenomena start to exist, although the major limiting factor is still diffusion-controlled. c) A highly depressed semicircle has started to develop, and no unambiguous inter-/extrapolation can be done, therefore an intermediate model is used. d) A more developed but still depressed semicircle reflects the prominent capacitive behavior of the sample. The equivalent circuit shown in the graph was employed to extract parameters for R_1, R_2, and CPE_1. In this model, the R_1 represents the resistance of the set-up, R_2 the bulk resistance of the studied material, and the CPE_1 the non-ideal capacitive behavior of the sample.

References

(1) Peresypkin, A. V.; Menger, F. M. Zwitterionic Geminis. Coacervate Formation from a Single Organic Compound, *Org. Lett.* **1999**, *1*, 1347–1350.

(2) Menger, F. M.; Peresypkin, A. V. A Combinatorially-Derived Structural Phase Diagram for 42 Zwitterionic Geminis, *J. Am. Chem. Soc.* **2001**, *123*, 5614–5615.

(3) Goossens, K.; Lava, K.; Bielawski, C. W.; Binnemans, K., Ionic Liquid Crystals: Versatile Materials, *Chem. Rev.* **2016**, *116*, 4643–4807.

(4) Martinelli, A.; Maréchal, M.; Östlund Å.; Cambedouzou, J. Insights into the interplay between molecular structure and diffusional motion in 1-alkyl-3-methylimidazolium ionic liquids: a combined PFG NMR and X-ray scattering study. *Phys. Chem. Chem. Phys.* **2013**, *15*, 5510–5517.

(5) Vranes, M.; Dozic, S.; Djeric V.; Gadzuric, S. Physicochemical Characterization of 1-Butyl-3-methylimidazolium and 1-Butyl-1-methylpyrrolidinium Bis(trifluoromethylsulfonyl)imide, *J. Chem. Eng. Data* **2012**, *57*, 1072–1077.
